# Dynamic Contact Angles on Moving Fibers Measured by X‑ray Holography

**DOI:** 10.1021/acs.langmuir.5c03213

**Published:** 2026-03-05

**Authors:** Louisa E. Kraft, Jens Lucht, Fiona Berner, Hannes P. Hoeppe, Tobias Eklund, Yizhi Liu, Markus Osterhoff, Fabian Westermeier, Wojciech Roseker, Tim Salditt, Hans-Jürgen Butt, Katrin Amann-Winkel

**Affiliations:** † 28308 Max Planck Institute for Polymer Research, Ackermannweg 10, 55128 Mainz, Germany; ‡ 9182 Johannes Gutenberg University, Institute for Physics, Staudingerweg 7, 55128 Mainz, Germany; § 9375 Georg-August-Universität Göttingen, Institut für Röntgenphysik, Friedrich-Hund-Platz 1, 37077 Göttingen, Germany; ∥ European XFEL, Holzkoppel 4, 22869 Schenefeld, Germany; ⊥ 28332 Deutsches Elektronen-Synchrotron DESY, Notkestrasse 85, 22607 Hamburg, Germany

## Abstract

Wetting of solid surfaces by a liquid is important for many natural and industrial processes, such as printing, painting, and coating. However, a quantitative description of the dynamic receding and advancing contact angle is still debated, in particular for aqueous solutions. One reason for our lack of quantitative understanding is the limited spatial resolution of currently used optical methods. We therefore present a new approach to access the submicroscopic region. We use X-ray phase contrast imaging to measure the dynamic receding contact angle on a moving glass fiber of 17 μm diameter. The fiber was pulled out of a liquid bath, which was filled with a mixture of glycerol and Milli-Q water. The dynamic receding contact angle decreased with increasing contact line velocity for all mixtures. In the holograms, we achieved a resolution of 50 nm/pixel with a spatial error of 450 nm. This spatial error is due to an extended surface region of the fiber and the liquid surface in the holograms. Our results demonstrate the feasibility of X-ray holography as a method to investigate dynamic contact angle phenomena and thereby open pathways to higher spatial and temporal resolution.

## Graphical abstract

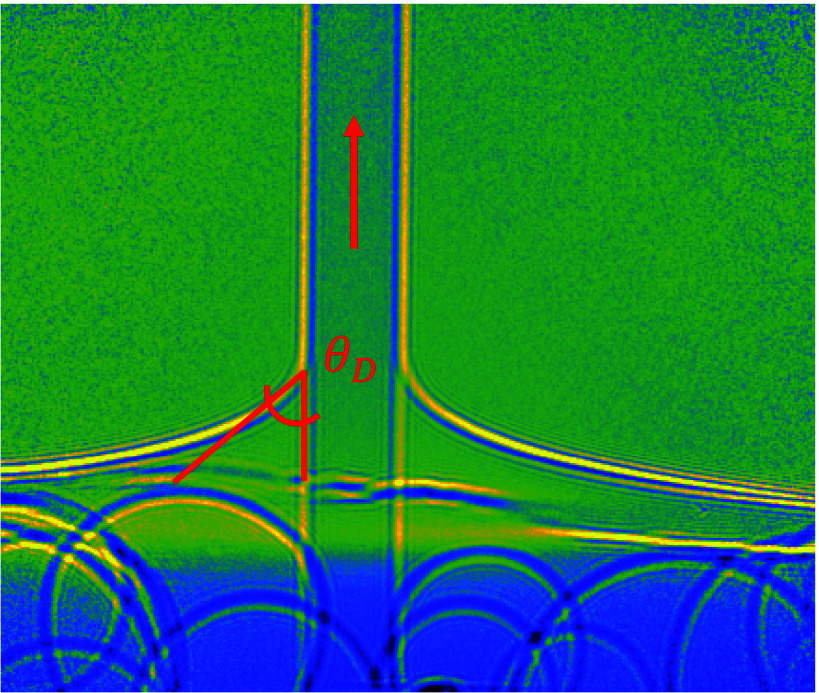

## Introduction

Wetting of solid surfaces is important for many industrial as well as natural processes, especially with respect to dynamical wetting. Examples include coating, painting, inkjet printing, heat exchangers, and flotation.^1^ When a liquid drop is deposited on a nonwetting or partially wetting surface, the contour of the liquid forms a finite contact angle in relation to the surface. A three-phase contact line forms at the interface of the vapor, the liquid, and the solid. Its tangent, the contact angle, is characteristic of the specific combination of vapor, solid, and liquid. In equilibrium, this contact angle is related to the involved interfacial free energies via Young’s equation.^2–4^ Experimentally, however, the equilibrium contact angle as stated in Young’s equation does not exist. Further, the solid–vapor and the solid–liquid interfacial energies cannot be measured. Instead, experimentally, a whole range of contact angles is detected for a specific solid–liquid combination, even on apparently homogeneous and smooth solids. The actually observed contact angle depends on how the wetting situation was created, e.g., how a drop was placed onto a surface. This range of possible contact angles is bounded by a lower and upper limit. The lower limit is the receding, the upper limit the advancing contact angle.^5,6^ This difference of the advancing and receding contact angle was termed ‘contact angle hysteresis’.^7^ Factors leading to contact angle hysteresis include roughness,^8^ heterogeneity,^9,10^ adaptation,^11^ slide electrification,^12^ and – on very soft surfaces – mechanical deformation.^13^ Contact angle hysteresis can therefore nowadays be used to characterize surfaces.

Significant progress has been made in terms of experimental characterization as well as understanding the static and macroscopic properties of liquid drops on solid surfaces, like the contact angle. However, certain aspects of the wetting dynamics of solid surfaces are still under debate. For example, the precise dynamic mechanism, with which the contact line advances across a solid, can thus far not be described quantitatively. A central observation in dynamic wetting is the velocity dependence of the dynamic advancing and receding contact angles. The dynamic advancing contact angle increases with increasing speed of the contact line, while the dynamic receding contact angle decreases with increasing velocity.^14^ Different models have been proposed to describe this velocity dependence.^15^ They assume different energy dissipation processes. The hydrodynamic model assumes viscous dissipation as the major source of energy dissipation. In the molecular kinetic theory (MKT), the advancing or receding contact lines have to overcome molecular energy barriers of the order of *k*_*B*_ *T*. In adaptation, it is assumed that the solid surface spontaneously changes once it is in contact with the liquid; energy is released during this relaxation process as heat. In slide electrification, charges are separated, and electrostatic work has to be carried out. The respective contributions of the different processes and the related theories are, however, still under debate.^16,17^ One example under current investigation is the role of surface defects.^18,19^ Recent research brought new insights into the influence of microstructures, such as topological and chemical defects, on contact line dynamics as well as droplet behaviors.^20–23^

Consequently, to date, no quantitative theory can predict such a simple event as a drop sliding down a tilted plate. This lack of understanding is largely caused by a lack of combined spatial and temporal resolution in microscopical methods.^24,25^ To understand dynamic wetting and to distinguish between different molecular processes, one would need to image the motion of contact lines with a resolution much better than 100 nm at a high frame rate. Usually, in experiments, the so-called macroscopic or ‘apparent’ contact angle is measured with a camera. From the videos, the contour of the liquid surface is extrapolated to the point of intersection with the solid surface.^26^ The method is fast, but the resolution is of the order of 10 μm. Using confocal microscopy, a resolution of the order of below 1 μm can be obtained,^27^ but the method is slow and limited to low speeds. Scanning electron microscopy (SEM) can achieve a higher resolution, but beam damage, low frame rate, and the presence of vacuum limit its applicability. In environmental SEMs, water drops can be imaged, but the resolution is not much better than in optical microscopy.^28,29^ Much better resolution can be achieved in atomic force microscopy, but imaging is challenging, the frame rate is low, and one can never be sure that the tip does not influence the shape of the liquid near the contact line.^30^ Further research uses reflection interference microscopy to visualize the microscopic contact line of droplets and the three-phase interfaces. The frame rate is hereby dependent on the camera, and therefore, high-speed imaging of the contact line is also possible.^31–34^

X-ray phase contrast imaging (XPCI) or X-ray holography using synchrotrons as an X-ray source can, in principle, overcome these limits. Few studies have already used X-ray imaging to investigate wetting in, for example, microchannels.^35,36^

In this work, we propose X-ray phase contrast imaging (XPCI) to compute high-resolution holograms of the dynamic receding contact angle with a spatial resolution of about 50 nm. Therefore, a moving glass fiber is pulled out of a liquid bath filled with aqueous glycerol mixtures. The use of X-rays instead of light microscopy promises an improved spatial resolution compared to common optical methods.

## Experimental Section

The measurements were taken at the ‘Göttingen Instrument for Nanoimaging with X-rays’ (GINIX), which is an endstation situated at the coherence applications beamline P10 at the PETRA III storage ring at Deutsches Elektronen-Synchrotron (DESY) in Hamburg.^37,38^ The GINIX endstation utilizes coherent X-ray radiation for propagation-based X-ray phase-contrast imaging (XPCI) for high-resolution imaging of the sample.^39–41^ In XPCI, phase shifts due to the sample interfere after sufficient, optics-free free-space propagation into measurable intensity patterns downstream at the X-ray detector. These resulting images are near-field diffraction patterns, or so-called (inline) holograms. A schematic depiction of the technical components can be seen in Figure 1a.

**Figure 1:**
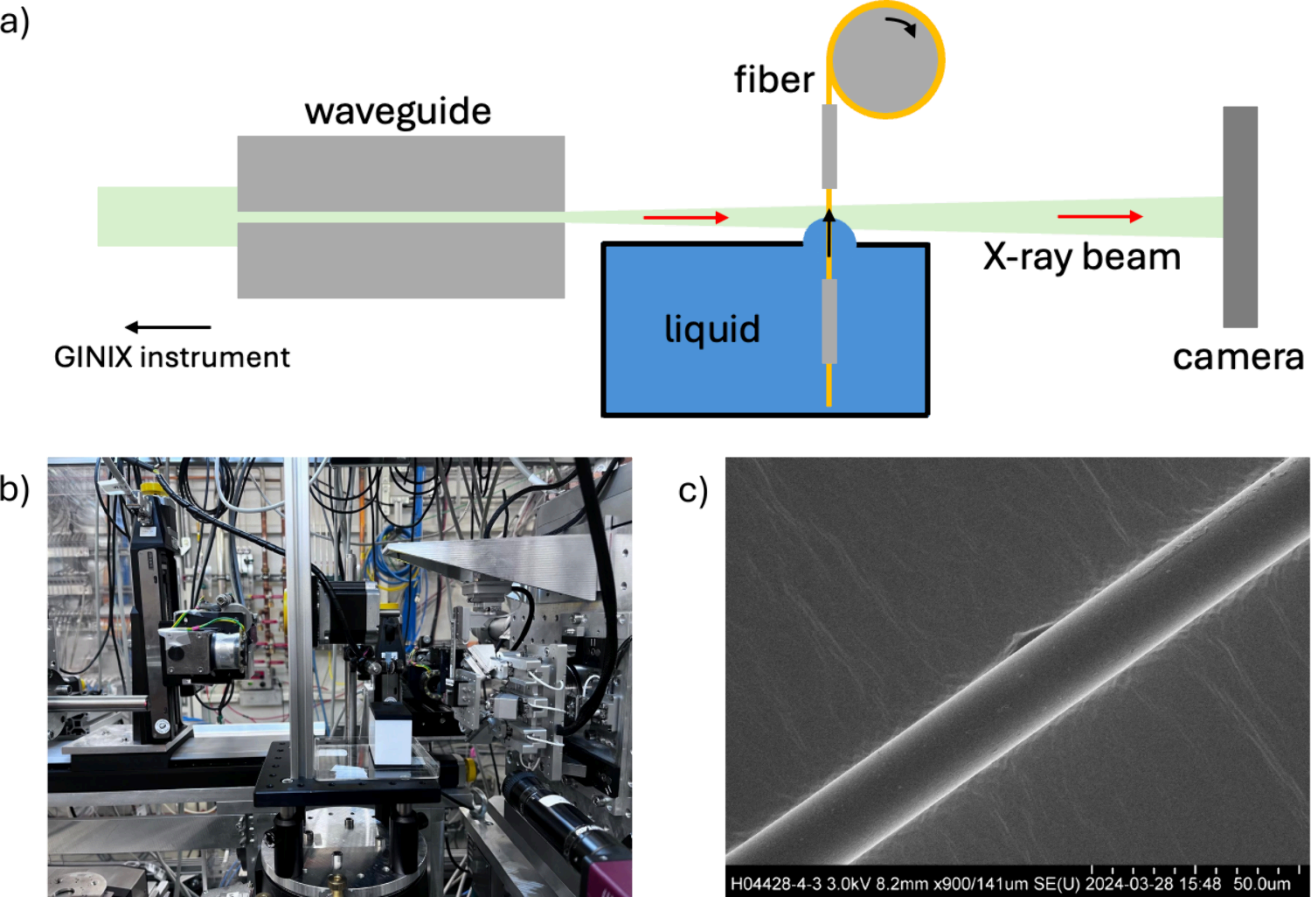
**a)** A schematic depiction of the experimental setup and of the waveguide configuration of the GINIX instrument at the P10 beamline at PETRA III, DESY, Hamburg. **b)** A picture of the sample stage. **c)** An SEM image of a cleaned glass fiber of 17 μm thickness.

The X-ray source was a 5 m long undulator. The X-rays passed a monochromator, which was a Si < 111> channelcut design, selecting an X-ray energy of 13.8 keV. The monochromator was followed by a set of slits and a set of mirrors in Kirkpatrick-Baez geometry, where the X-rays were focused in vertical and horizontal directions. At the focal point, the beam spot size was about 300 nm x 300 nm. The waveguide source, which was situated in the focal point, acted as a quasi-point source and a low-pass filter to reduce high-frequency artifacts in the holograms. It was formed by two crossed lamellae of planar waveguides formed by a thin film sequence of Mo/C/Mo with a guiding layer thickness of 80 nm.^42^ The sample stage (Figure 1b) was situated slightly behind the focus in the diverging beam. Thus, by the Fresnel scaling theorem the placement of the sample resulted in a field of view of about 160 μm in both horizontal and vertical directions in the final holograms, corresponding to an effective pixel size of about 61 nm and geometric magnification of 122. After illumination with the cone-shaped beam, the X-rays were collected at a distance of 5 m. Images were detected using a Zyla sCMOS camera with a fiber-optic plate detector (2560 × 2160 pixels with a respective physical pixel size of 6.5 μm x 6.5 μm).

The sample consisted of a 17 μm thick glass fiber, which was pulled vertically out of a liquid bath by a programmable motor with a defined velocity in a range between 0 and 5 mm/s. Hereby, a 2-phase bipolar stepper motor from Oriental Motor was used. The glass fiber was additionally secured in its position with two syringes, minimizing the horizontal movement of the fiber in relation to the beam. The liquid bath (a customized Teflon box) was closed with a lid, which was hydrophobically coated with Glaco spray (Glaco Mirror Coat Zero). The lid had a hole of 1 cm in diameter. A liquid dome was formed in the hole by slightly overfilling the liquid bath. This reduction of the liquid surface area ensured that the slightly inclined X-ray beam would not be clipped at the edge of the bath while measuring the liquid surface and the meniscus as close as possible. As liquid, we used Milli-Q water-glycerol mixtures of 30, 50, and 80 wt % glycerol (98% level of purity from Fisher Scientific). Before measuring the fibers were immersed in the liquid bath for several minutes until the pull-up process was set up. The glass fibers (Hybon 2002 fibers by Nippon Electric Glass) were precharacterized by scanning electron microscopy (SEM, FEI Nova 600 NanoLab) (Figure 1c). Before inserting the fibers into the setup, they were cleaned in toluene.

## Results and Discussion

For each aqueous glycerol mixture and fiber speed, we obtained holograms. A hologram of a fiber can be seen in Figure 2a. It shows the fiber and the meniscus of the liquid. In the respective example, an 80 wt % glycerol–water mixture has been used, and the fiber was pulled upward with a velocity of 1 mm/s. The air bubbles seen in the hologram were generated when mixing glycerol and water and further pouring the mixture into the liquid bath. However, the bubbles did not interfere with the liquid surface close to the fiber. As a result of the imaging method, there is no further information on where exactly in relation to the fiber the bubbles were positioned, as there is no depth of field in the holograms. We can exclude, however, that they were in the meniscus.

**Figure 2:**
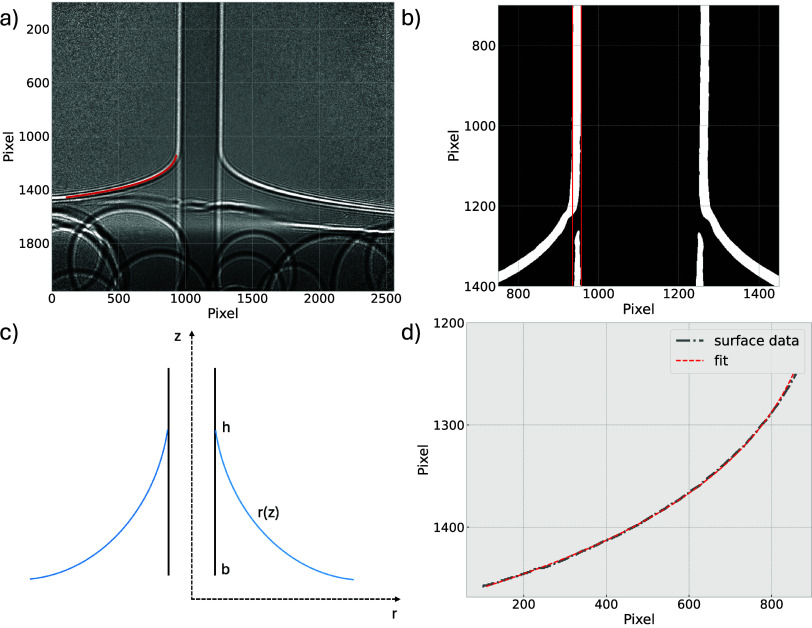
**a)** Hologram of a glass fiber pulled upward with a velocity of 1 mm/s in an 80 wt % glycerol–water mixture. The red line represents the fit of the liquid surface (see also d). **b)** A zoomed-in image of the glass fiber. The spatial error in the hologram is based on the extended fiber surface region, indicated by the two red lines. **c)** Schematic depiction of the meniscus on a vertical fiber, including all parameters of the model described by eq 1. **d)** Fit of the liquid surface.

The analysis was performed using the HoToPy Python toolbox for XPCI.^43^ To determine the spatial error in the obtained holograms, the central fiber is magnified (Figure 2b). The two red lines indicate the fiber surface region, i.e., the point of change in contrast. In the obtained holograms, we achieved a spatial resolution of 50 nm/pixel with a spatial error of 450 nm. This error is due to this extended surface region of the fiber, which amounts to more than one pixel in width. The exact placement of the real fiber surface within this region is ambiguous, resulting in a spatial error of 450 nm, which is half the length of the extended fiber surface region. The same spatial error could be observed for the interfacial region of the liquid surface.

The imaged liquid surface, or meniscus, on the vertical fiber can be described by solving the Laplace equation in radial coordinates.^44^ In the holograms, the field of view corresponds to a length of 160 μm. Within this regime, which is smaller than the capillary length, the capillary force surpasses the gravitational force, hence, we neglected gravity in our model. The meniscus can then be described using the following equation:^45^

1 $$r\left(z\right)=b\;\cosh\left(\frac{z-h}{b}\right)$$

where *h* is the height measured at the three-phase contact point and *b* is the radius of the fiber. A schematic depiction of the meniscus with all relevant parameters is displayed in Figure 2c.

The measured liquid surface can be fitted using the model described in eq 1 (Figure 2d). The shown data corresponds to the measurement of 80 wt % glycerol in Milli-Q water in Figure 2a. The model of the liquid meniscus sufficiently describes the curvature of the liquid surface. The quality of the fit can also be seen when the fit is plotted in the hologram (Figure 2a, red line; see also Figure S.1 for data analysis workflow).

Each measurement cycle for each solution and constant velocity consisted of the same sequence. First, the X-ray beam was focused on a position where neither the glass fiber nor the liquid surface was visible, to collect empty background images. These are needed to compute the holograms, for which the intensity in the sample image is divided by the intensity in the background image. Then, the beam position was changed to focus on the liquid meniscus and the glass fiber. In this configuration, 200 images were taken. 50 images with a static, nonmoving fiber (Figure 3a) followed by 100 images with a moving fiber (Figure 3b), and finally again 50 images with a static fiber after the motor was stopped (additional example provided in Figure S.2). The exposure time for every image was 40 ms. Figures 3a and 3b show single holograms. The example shows data from an 80 wt % glycerol–water mixture, where in the dynamical case (Figure 3b), the glass fiber was pulled upward with a velocity of 1 mm/s.

**Figure 3:**
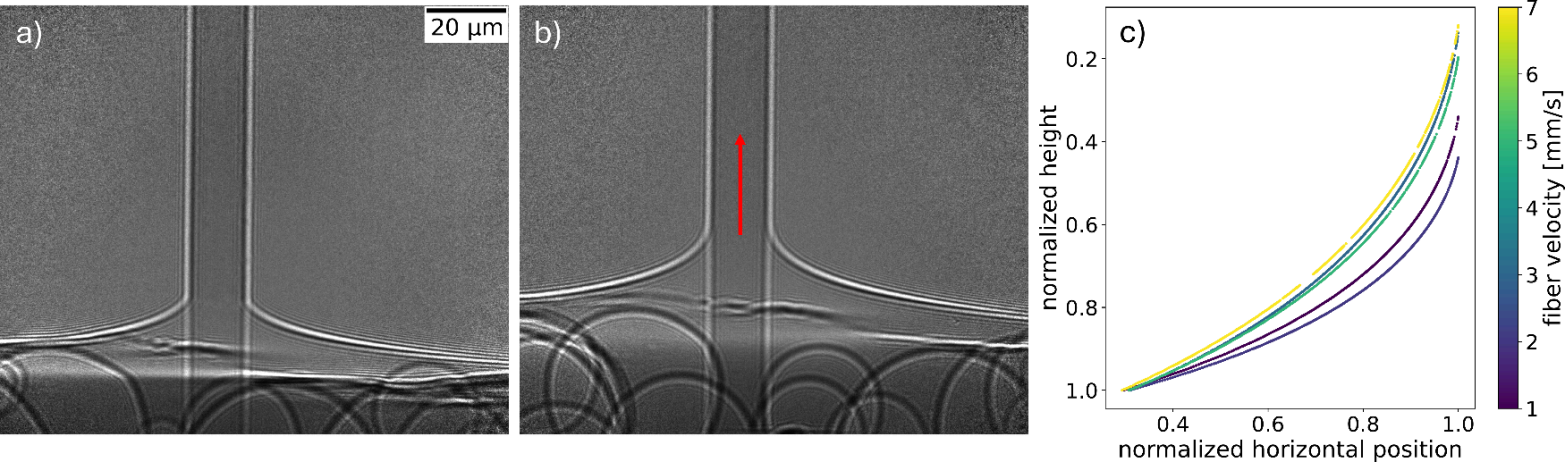
Hologram series of a measuring cycle for 80 wt % glycerol in Milli-Q water: **a)** static fiber, **b)** moving fiber with a velocity of 1 mm/s. The red arrow indicates the direction of movement of the glass fiber. **c)** The liquid meniscus plotted for different fiber or contact line velocities for 80 wt % glycerol in Milli-Q water. The plotted menisci stem from the same run; hence, the fiber was not changed while varying the velocity.

The edges of the fiber, as well as the liquid, were extracted by scanning the image, respectively horizontally and vertically, and identifying the pixel positions corresponding to a jump in contrast (thresholding). The pixel positions associated with the liquid meniscus were fitted using the model described in eq 1. In Figure 3c, the curvature of the liquid meniscus is plotted for different fiber velocities (i.e., contact line velocities) ranging from 1 mm/s (blue) to 7 mm/s (yellow). In order to directly compare the different curvatures, the baseline of the liquid surface was normalized and set to the same vertical position. The measured heights are hereby all divided by the value of the lowest vertical position of each curve, which corresponds to a higher pixel number. For this reason, when the real height increases, the value of the normalized height in the plot decreases. All curvatures start at the baseline value of 1. This is necessary to ensure the curvatures are visually comparable. Within the 160 μm field of view, the whole imaged liquid surface surrounding the glass fiber is pulled upward by the movement. The surface does not recede back to its equilibrium position within the field of view. The baseline of the liquid surface surrounding the fiber is therefore higher for higher contact line velocities.

Essentially, the same correction is applied to account for the horizontal movement of the fiber between or during measuring cycles. The edge of the fiber surface is hereby set to the same position. The surface of the glass fiber was situated at the value of 1.

The highest point of the liquid meniscus, which is directly on the fiber surface, corresponds to the three-phase contact point. The highest vertical position of the three-phase contact point is reached for the highest velocity. The liquid is dragged upward with the glass fiber, affecting the curvature of the meniscus (Figure 3c).

Three solutions with different glycerol fractions, and hence different viscosity, have been measured (Figure 4). The analysis, i.e., the computation of the dynamic receding contact angle, was done using single holograms of the moving or static fiber. These holograms were analyzed to determine the dynamic receding contact angle on the moving glass fiber. The angle was calculated within a region of 500 nm from the three-phase contact point by applying a tangent to the liquid surface in this region. For this tangential fit, always 10 pixels were used. The dynamic contact angles can then be calculated using the angle or slope of the tangential fit of the liquid surface and the slope of the linear relation describing the fiber.

**Figure 4:**
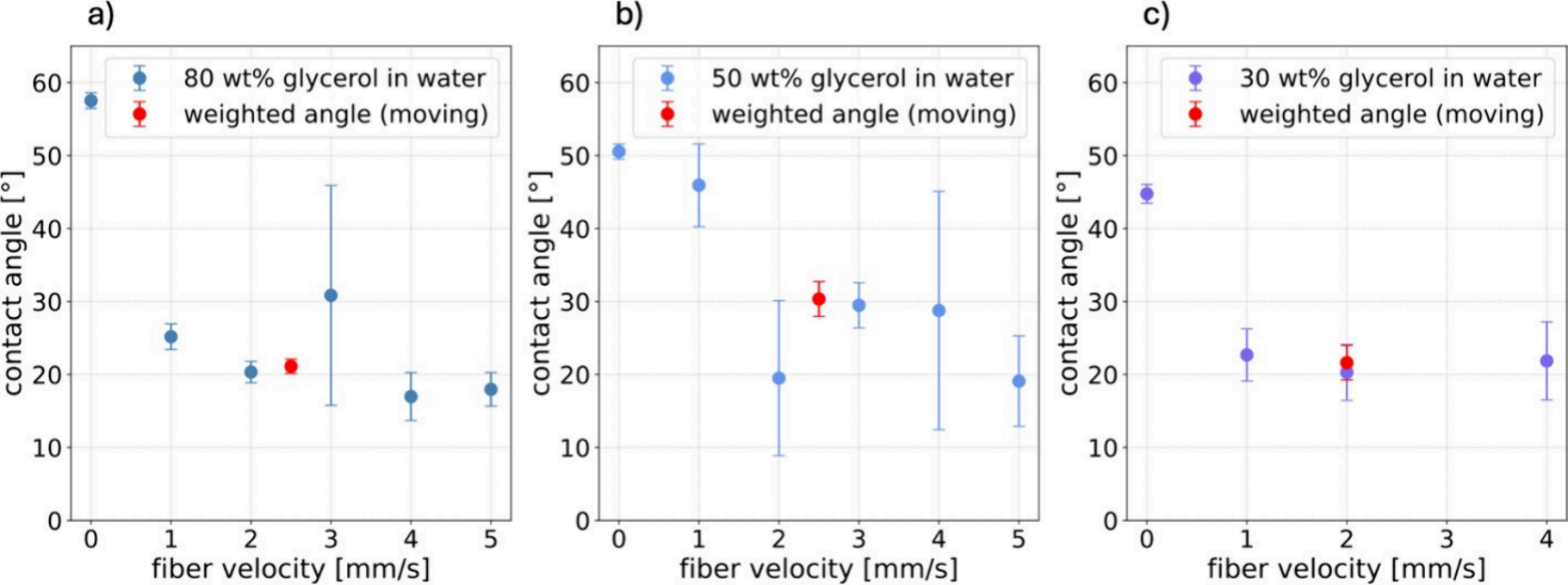
Dynamic receding contact angle in dependence on the fiber velocity for **a)** 80 wt %, **b)** 50 wt %, and **c)** 30 wt % glycerol in Milli-Q water. In red, the weighted average angles for the moving fibers are shown.

The dynamic receding contact angles tended to decrease with increasing speed of the contact line or fiber (Figure 4). This could be observed for all three aqueous glycerol mixtures. For each respective fiber speed, five different holograms were analyzed. The dynamic receding contact angle on the glass fiber was then taken to be the mean value, averaging over those five measurements. The error is the standard deviation of this value. The contact angle calculations were done for measurements in a velocity range between 0 mm/s, corresponding to a static fiber, and 5 mm/s. Neither the hydrodynamic nor the MKT model fit the results properly (see also Figure S.3). We therefore believe other processes dissipate energy. One such process is charge deposition and the resulting increase in solid surface energy.^46^ In addition, the solid surface may have adapted while being immersed in the liquid and the reversal back to the dry state may take time, leading to a speed-dependent effective solid surface energy. Further investigation in future experiments is needed in this regard.

The biggest change in the value of the dynamic receding contact angle can be observed by comparing the contact angle of the static glass fiber (0 mm/s) and the weighted average contact angle for the cases of moving fibers, from 1 to 5 mm/s. This is true for all three mixtures. The weighted average angles are additionally shown in Figure 4 (red dots).

The largest contact angle of around 58° for a static glass fiber was measured for the mixture of 80 wt % glycerol in water. The static angle decreased with a decreasing wt % of glycerol in water, hence decreasing viscosity (see Table S.1). Large standard deviations, i.e., errors in the contact angle analysis, stem from horizontal movement and vibrations of the fiber. The vibrations were mostly caused by the setup itself. The horizontal movement of the fiber was within the small range of about half the field of view, about 80 μm, because the syringes allowed some movement space. Both influence the overall quality of the holograms. Horizontal fiber movement as well as vibrations could be especially observed for larger velocities, those exceeding 5 mm/s. Those velocities are therefore not presented in the analysis above.

## Conclusion

X-ray phase contrast imaging was demonstrated to be a feasible method to investigate dynamic wetting phenomena. Using this method, we could obtain holograms of a static and dynamic contact line by pulling a 17 μm thick glass fiber out of a liquid bath. Hereby, three different glycerol–water mixtures with varying viscosity have been studied. When the fiber is pulled upward, the entire liquid meniscus is elevated within the field of view of 160 μm. The shape of the meniscus is usually determined by the equilibrium between capillary forces and gravitational forces. Since our field of view was much smaller than the capillary length, gravity can be neglected. The liquid surface in the holograms can be fitted with the corresponding Laplace equation. The dynamic receding contact angle decreases from around 50° to 20° with increasing contact line velocity (1–5 mm/s) for all three mixtures. We were able to determine the dynamic receding contact angle on moving glass fibers within a region of 500 nm measured from the three-phase contact point. In the holograms, we achieved a spatial resolution of 50 nm/pixel with a spatial error of 450 nm. We propose that X-ray phase contrast imaging can be used in future experiments on wetting dynamics. Higher temporal and spatial resolution could potentially be achieved using an X-ray free-electron laser.

## Supplementary Material



## Notes

Information (graphical form) on the data analysis workflow, as well as further images highlighting the measurement cycle and the symmetry of the meniscus on the fiber, especially for the static case (graphical form); a figure where the hydrodynamic theory is applied to the measured data (graphical form) and a table showing dynamic viscosity measurements of our liquid samples (Table S.1) (PDF)
