# Supplementary material for: Dynamic Contact Angles on Moving Fibers Measured by X‑ray Holography
Source: Langmuir. 2026 Mar 5;42(15):10180–6. doi: 10.1021/acs.langmuir.5c03213 (PMC13104168; doi:10.1021/acs.langmuir.5c03213)
Supplement: Supplementary file 1 [file la5c03213_si_001.pdf]

Dynamic contact angles on moving fibers measured by X-ray holography

Louisa E. Kraft[1,2], Jens Lucht[3], Fiona Berner[1,2], Hannes P. Hoeppe[3], Tobias Eklund[1,2,4], Yizhi Liu[1], Markus Osterhoff[3], Fabian Westermeier[5], Wojciech Roseker[5], Tim Salditt[3], Hans-Jürgen Butt[1], Katrin Amann-Winkel[1,2,*]

* amannk@mpip-mainz.mpg.de

[1]Max Planck Institute for Polymer Research, Ackermannweg 10, 55128 Mainz, Germany

[2]Johannes Gutenberg University, Institute for Physics, Staudingerweg 7, 55128 Mainz, Germany

[3]Georg-August-Universität Göttingen, Institut für Röntgenphysik, Friedrich-Hund-Platz 1, 37077 Göttingen, Germany

[4]European XFEL, Holzkoppel 4, 22869 Schenefeld, Germany

[5]Deutsches Elektronen-Synchrotron DESY, Notkestrasse 85, 22607 Hamburg, Germany

Supporting Information

Data analysis workflow

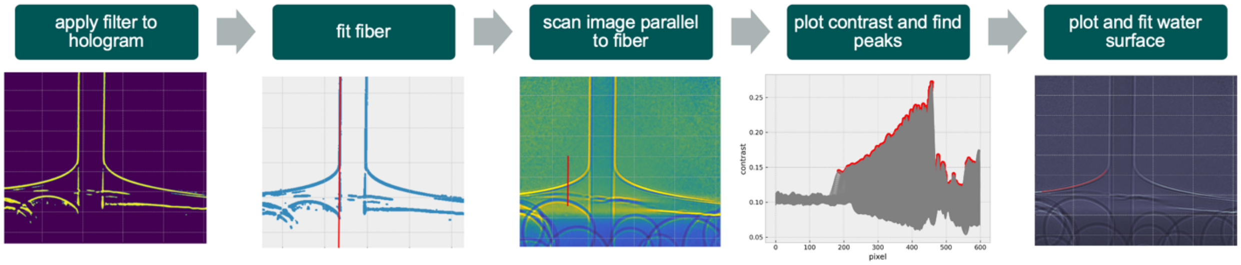

Figure S.1. *Graphical representation of the image processing of a single hologram, shown as an example for 80 wt% glycerol in water. First, a filter is applied to the hologram to identify the interfacial information. In the next step, the fiber is fitted using a linear relation. The surface area is scanned parallel to the fiber, and the contrast peaks are identified. The positions of the highest contrast adhere to the positions of the liquid surface in the image. Finally, the liquid surface is fitted.*

Measurement cycle

The following figure shows another measurement of the glass fiber pulled upwards while the liquid bath is filled with 80 wt% glycerol in Milli-Q water. The images, especially the two holograms depicting the static fiber, should underline the symmetry of the liquid meniscus on both sides of the fiber. The air bubbles did not interfere with the liquid surface close to the fiber, as no significant differences could be seen in the measurements with more bubbles compared to those with fewer.

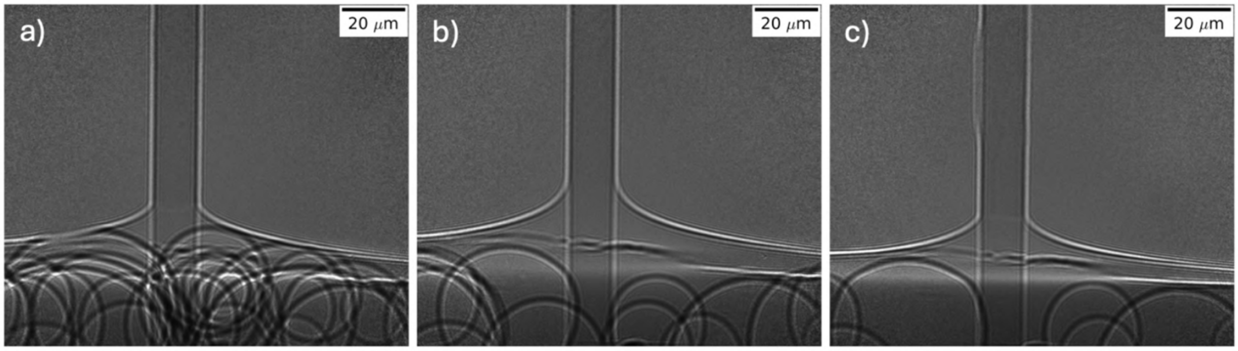

Figure S.2. *A series of images representing one measurement cycle:* ***a)*** *a static fiber,* ***b)*** *a moving fiber with a velocity of 3 mm/s,* ***c)*** *a static fiber. The bath is filled with 80 wt% glycerol in Milli-Q water.*

Applying hydrodynamic theory

Different models have been proposed to describe the velocity dependence of the dynamic contact angle. We here tried to fit Voinov's model [1], which is described by the following relation:

$$\theta_D^3(v) = \theta_0^3 + \frac{9\eta v}{\gamma} \cdot \ln\left(\frac{L}{L_m}\right),$$

with the dynamic θ_D and static contact angle θ_0, the dynamic viscosity η, the surface tension γ, and the microscopic L_m and mesoscopic length scale L. The data, however, are not well represented by this model in all three cases.

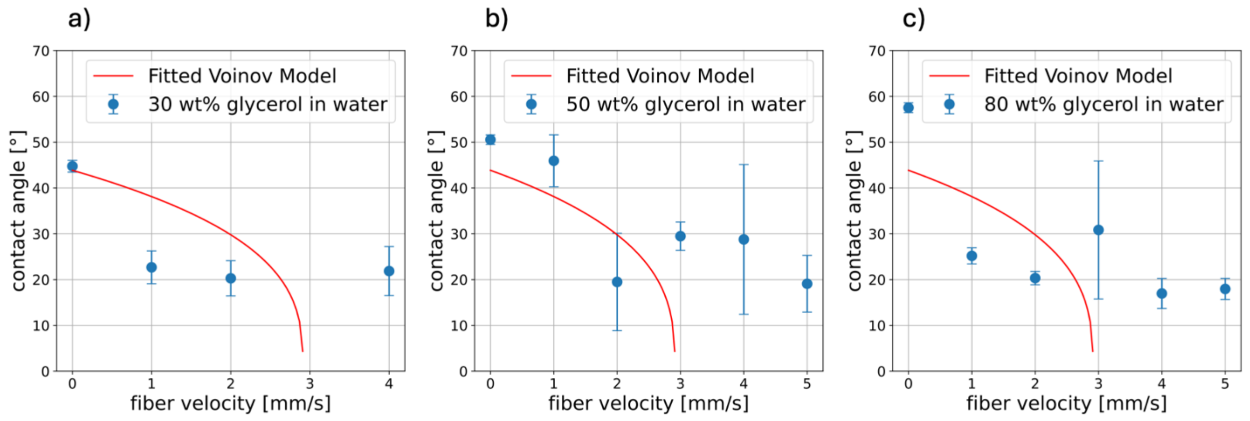

Figure S.3. *Hydrodynamic theory applied to the measured data of the dynamic receding contact angle for* ***a)*** *30 wt%* ***b)*** *50 wt%* ***c)*** *80 wt% of glycerol in Milli-Q water. The fit function is described by the following relation:* $\theta_D^3(v) = \theta_0^3 + \frac{9\eta v}{\gamma} \cdot ln\left(\frac{L}{L_m}\right)$, *where the natural logarithm is treated as one variable A.*

[1] Voinov, O. V. Hydrodynamics of wetting. *Fluid Dyn* **1977**, *11*, 714–721.

Dynamic viscosity of samples

Table S.1. *Dynamic viscosity at 20°C for the used glycerol-Milli-Q water mixtures. The measurements were done using the Lovis 2000 M/ME Rolling-Ball viscometer from Anton Paar.*

**Sample**	**80 wt% glycerol in Milli-Q water**	**50 wt% glycerol in Milli-Q water**	**30 wt% glycerol in Milli-Q water**
**Dyn. Viscosity [mPa·s]**	55.8	5.6	2.4
**Deviation [%]**	0.08	0.04	0.22
